# The Impact of Financial Deepening on Carbon Reductions in China: Evidence from City- and Enterprise-Level Data

**DOI:** 10.3390/ijerph191811355

**Published:** 2022-09-09

**Authors:** Kai Tang, Qianbo Chen, Weijie Tan, Yi Jun Wu Feng

**Affiliations:** School of Economics and Trade, Guangdong University of Foreign Studies, Guangzhou 510006, China

**Keywords:** financial deepening, carbon emissions, green innovation, China

## Abstract

This study extends the limited evidence of the China context by establishing a panel fixed-effect model to identify the nexus between financial deepening and carbon emissions. Using newly compiled city-level (287 prefecture-level and above cities) and enterprise-level (resource enterprises listed on the Chinese A-shares) datasets from 2007 to 2019, this study quantitatively evaluated finance deepening and analysed the impact of financial deepening on carbon emissions in China, with a particular consideration of green innovation. Our results document that financial deepening contributes to carbon reductions, as shown by the considerably decreased carbon dioxide (CO_2_) emissions. Both the city-level and enterprise-level estimates argue that financial deepening has a promoting effect on green innovation. Stimulating green innovation is identified as an important mechanism through which financial deepening can contribute to carbon reductions. Policy implications are presented based on the empirical results.

## 1. Introduction

To limit global warming to well below 2 °C and limit the temperature increase to 1.5 °C above pre-industrial levels, the global community needs to reduce carbon emissions substantially and achieve net-zero emissions as soon as possible [1,2,3]. For many developing economies, effective carbon reductions have become a prime importance of policymakers. A typical example is China, the world’s largest developing economy and top carbon emitter [4,5]. However, most developing economies, including China, are still in their industrialization and urbanization process, which means that the demand for energy and the associated carbon emissions are highly likely to continue increasing [6,7,8,9]. These are considered challenging to reduce carbon emissions substantially in those economies.

Concurrently, China, as the world’s largest developing economy and top carbon emitter, is facing institution changes in the process of industrialization and urbanization. Many of the changes are the results of marketization and economic growth [10,11,12,13]. One change that has received increasing scholarly attention is the deepening financial systems [14,15,16,17]. Financial deepening, defined as the increased provision of financial services, is widely believed to bring in a better ability to mobilize resources, allocate funding and diversify risks [18], thus contributing to innovation, economic growth, urban–rural integration and social welfare improvement [19,20,21]. Moreover, financial deepening is also one of the key characteristics of China’s urban–rural integration [22,23]. Specifically, a typical character of financial deepening in China is the expansion of bank presence [24,25,26].

China’s urban–rural development, partially characterized by financial deepening, is likely to have an impact on changes in the ecological environment such as massive carbon emissions. The dominant view in the literature is that financial deepening contributes to economic growth [27,28,29]. Evidence from multiple countries has shown that this positive nexus may cause enterprises to expand their output and result in the increase of associated carbon emissions [30,31,32,33]. However, several recent studies have presented different empirical results and argue that financial deepening might promote the financing of producers and the adoption of low-emissions technologies, thus decreasing emissions [34,35]. So, the overall effect of financial deepening on carbon emissions is uncertain and thus requires further evidence. Besides, empirical research involving the impact of financial deepening on carbon emissions in China is still relatively rare. Such evidence will help in understanding the impact channels and make better policy impact evaluation.

China provides excellent opportunities to understand the impact of financial deepening on carbon emissions. Since the mid-1990s, there has been a profound change in China’s financial institution, characterized as less regulated and more market-driven [25,26,36]. China’s financial reform accelerated, weakening local governments’ control over local branches of state-owned banks. Besides, the central government began to allow the establishment of local commercial banks. Moreover, some foreign banks have been allowed to expand their branches to China’s cities. These actions promote the competition in local financial markets and weaken the monopoly of large state-owned financial institutions, thus increasing provision of financial services and easing the financial constraints of enterprises to a certain extent. At the same time, China’s carbon emissions have continuously been increasing. However, the pace of emissions growth has moderated recently (an average annual rate of 9% in the 2000s and 3% in the 2010s) [37], and some argued that China’s carbon emissions have plateaued [38].

In this context, this study aimed to address the effect of financial deepening on carbon emissions in China, with a particular consideration of green innovation. We find that financial deepening contributes to carbon reductions, as shown by the considerably decreased CO_2_ emissions. Promoting green innovation is identified as an important mechanism through which financial deepening can contribute to carbon reductions. The 2SLS method is adopted to effectively solve the endogenous problem. These results are robust across a serious of robustness checks.

This study contributes to the existing literature in the following ways. First, it extends the limited literature of the China context by identifying the nexus between financial deepening and carbon emissions empirically. Second, this study used newly compiled city-level (287 prefecture-level and above cities) and enterprise-level (resource enterprises listed on the Chinese A-shares) datasets. Such large amounts and multiple levels of observations will not only help us to obtain more robust estimates, but also help us explore how the nexus between financial deepening and carbon emissions is affected by the urban and enterprise characteristics. Third, this study includes green innovation in the empirical framework, thereby enhancing the understanding of the finance–development nexus.

The rest of this paper is organized as follows. In Section 2, we briefly conduct a theoretical analysis. In Section 3, we present the methods and data, followed by an empirical analysis in Section 4. Finally, Section 5 provides the conclusions.

## 2. Theoretical Analysis

### 2.1. The Effect of Financial Deepening on Carbon Emissions

Theoretically, there are two opposing views in terms of the effect of financial deepening on carbon emissions. On one hand, financial deepening fuels China’s industrialization and economic growth through reducing the credit constraints and enabling enterprises to have access to capital with less costs [27,39]. Enterprises have an incentive to expand their production, which may result in the increase of energy use and more carbon emissions [32,33]. Moreover, financial deepening may also cause consumers to have access to cheap credits, which will stimulate consumption and the associated energy use [40,41]. Consequently, financial deepening may increase China’s carbon emissions.

On the other hand, the relatively cheaper capital as a result of financial deepening could better meet the funding needs of updating technologies, thus increasing the possibility of adopting cleaner and more sustainable production technologies [33]. Besides, financial deepening may also attract foreign direct investment (FDI), which is usually accompanied by knowledge spillovers [42], an effective channel for green technology transfer [43,44]. Therefore, energy efficiency in China is likely to be enhanced due to green technology transfer, thereby achieving carbon reductions. In addition, financial deepening could strengthen the positive connection between renewable energy development and carbon reductions [45]. Accordingly, financial deepening may result in carbon reductions.

In summary, the effect of financial deepening on carbon emissions in China is still uncertain. Hence, this study proposes two hypotheses:

**Hypothesis** **1a.** *Financial deepening results in increased carbon emissions in China.*

**Hypothesis** **1b.** *Financial deepening contributes to China’s carbon reductions.*

### 2.2. The Channel of Financial Deepening Impacting Carbon Emissions

There are multiple factors that might affect the impact of financial deepening on carbon emissions. Among them, green innovation is likely to play a key role. In literature, there is a growing consensus about the nexus between green innovation and carbon emissions; green innovation has a positive effect on carbon reductions [46,47,48]. However, there are different theoretical arguments on the relationship between financial deepening and green innovation.

The majority of relevant literature supports that financial deepening has a positive effect on green innovation [49,50,51]. Given the long investment return period and high uncertainty, green innovation usually needs continuous and considerable amounts of investment [52,53]. Hence, financial deepening could promote the financing of green innovation to effectively meet the capital need. Moreover, financial deepening usually brings about cheaper credit and capital with less costs [29,54], which could reduce the cost of green innovation. In this context, enterprises are more likely to engage in green innovation.

Contrarily, several studies argue that financial deepening may hinder green innovation. Cross-country evidence has shown that financial deepening makes the credit market grow and mature, which may be accompanied by financial providers’ growing risk aversion [55]. As a result, a mature credit market may discourage green innovation, which is widely characterized as high risk, due to the growing risk aversion and other considerations. Besides, Lv et al. (2021) [56] stated that financial deepening in terms of scale expansion makes the financial system more complicated, which may cause decreased stability and more difficulty in managing funds. When a financial institution is overexpanded, its efficiency may decrease, which may constrain enterprises’ innovation investment, since the required credit could not be provided effectively. In this case, green innovation will subsequently be negatively impacted.

In summary, green innovation contributes to carbon reductions, while the effect of financial deepening on green innovation is still uncertain. Hence, this study proposes another two hypotheses:

**Hypothesis** **2a.** *Promoting green innovation is an important mechanism through which financial deepening can contribute to carbon reductions.*

**Hypothesis** **2b.** *Hindering green innovation is an important mechanism through which financial deepening results in increased carbon emissions.*

## 3. Methodology and Data

### 3.1. Model

To examine the impact of financial deepening on carbon emissions in China, the following econometric model is first proposed:

*co*2*_it_ = α*_1_*+ β*_1_*FD_it_ +γ**X** + Year + City + **u*** (1)

where *co*2*_it_* represents carbon emissions (log value) for city *i* in year *t*; *FD_it_* denotes city-level financial deepening; ***X*** is a vector of control variables; *Year* and *City* represent year- and city-fixed effects, respectively; and ***u*** is the error term.

Then, we run the following regression to gauge if financial deepening influences green innovation:

*green_it_ = α*_2_*+ β*_2_*FD_it_ +γ’**X** + Year + City + **u**’* (2)

where *green_it_* represents the green innovation level for city *i* in year *t*; ***X*** is a vector of control variables; and ***u***’ is the error term. Others are the same as in Equation (1).

The previous estimates are based on city-level data. In China, resource enterprises have been identified as a major carbon emitter in cities [57,58,59,60]. Therefore, it is useful to explore the effect of financial deepening on carbon emissions with a consideration of green innovation using resource enterprises’ data.

The enterprise-level empirical strategy is similar to the above city-level analysis. Since the carbon emissions data of China’s resource enterprises are unavailable, we therefore empirically explore the role of green innovation in the identified nexus. Specifically, the following regression is proposed to gauge if financial deepening influences green innovation:

*patent_j,t+_*_1_*= α*_3_ *+ β*_3_ *bank_jt_ +δ**Y** + Year + Industry + **v*** (3)

where *patent_j,t+_*_1_ represents the green innovation level for resource enterprise *j* in year *t* + 1; We use *t* + 1 to address the potential endogenous issue as suggested. Besides, the impact of the financial institution change on carbon emissions at the enterprise level may also be lagged, which also supports the use of *t* + 1. *bank_jt_* denotes enterprise-level financial deepening; ***Y*** is a vector of control variables; *Year* and *Industry* represent year- and industry-fixed effects, respectively; and ***v*** is the error term.

### 3.2. Methods

The panel fixed-effect model is adopted for econometric analysis. Its advantages are mainly as follows. First, the panel data permit more complicated analyses compared to cross-sectional data since they reduce the possibility of collinearity among variables and enhance the validity of estimation results [61]. Second, the panel fixed-effect model controls for time-invariant omitted terms and allows them to freely correlate. For the purpose of testing the reliability of the empirical estimates, a series of robustness tests are also conducted. We first replace the core variables and include the regional heterogeneity. Then, we also consider the joint impact with other policies and the possible lagged effects. Besides, the endogenous test is run by using the instrument variable approach to estimate the reverse causality that cannot be solved by the used models [34,62,63].

### 3.3. Variables and Data

#### 3.3.1. City-Level Variables

This study compiles a new city-level dataset including 287 prefecture-level and above cities in China’s mainland from 2007 to 2019, with the consideration of data availability. Carbon emissions are measured using the logarithmic value of city carbon dioxide emissions (*co2*). City-level carbon dioxide emissions data are from China Emission Accounts & Datasets (CEADs) [64]. According to Bernini and Brighi (2018) [62] and Hasan et al. (2017) [65], city-level financial deepening is proxied by the density of bank branches (number of Chinese and foreign bank branches to the size of the city) (*FI*). City-level bank branches data are obtained from the financial license information database of the China Banking Regulatory Commission (https://xkz.cbirc.gov.cn/jr/, accessed on 4 March 2022). Following Berrone et al. (2013) [66] and Consoli et al. (2016) [67], the city green innovation level is measured by the number of green patents per 100 employees. The city-level population and green patents data are obtained from the *China Urban Statistical Yearbook* (2008–2021) and the China National Intellectual Property Administration (https://pss-system.cponline.cnipa.gov.cn/conventionalSearch, accessed on 15 March 2022), respectively. Specifically, green patents are identified as those that fall within the scope of the International Patent Classification (IPC) Green Inventory (https://www.wipo.int/classifications/ipc/en/green_inventory, accessed on 20 March 2022).

As for control variables, this study considers the following ones which may influence financial deepening and/or carbon emissions: the level of economic development (*pgdp*; measured by urban GDP per capita), population size (*pop*; measured by the logarithmic value of population), the level of industrialization (*ind*; measured by the ratio of the secondary industry output value to GDP), the level of urbanization (*urban*; measured by the ratio of urban area population to the city’s total population), energy structure (*es*; measured by the coal share of total primary energy supply), wage level (*citywage*; measured by the logarithmic value of the average salary of the city’s employees), and consumption level (*rsc*; measured by the logarithmic value of the total retail sales of social consumer goods) [36,68,69,70]. The data used for measuring those controls are from the *China Urban Statistical Yearbook* (2008–2020). Monetary items are measured in 2007 constant prices. The details measurements and descriptive statistics of variables are shown in Table 1. In addition, all the variables mentioned above are measured using annual data.

#### 3.3.2. Enterprise-Level Variables

This study compiles a new enterprise-level dataset including resource enterprises listed on the Chinese A-shares from 2007 to 2019 with the data availability consideration. The resource enterprises’ basic information and financial data are sourced from the China Stock Market & Accounting Research (CSMAR) Database and the Wind Economic Database, and enterprises’ patent data come from the State Intellectual Property Office of the People’s Republic of China. Enterprises that have suffered data losses are excluded. Following Chay et al. (2015) [71], we also sort the continuous variables and winsorize the 1% and 99% quantiles to reduce the outliers’ impact.

Inspired by Beck et al. (2019) [72], Brown et al. (2016) [73] and Skrastins and Vig (2019) [74], geographical proximity is employed to measure financial deepening at the enterprise level, which is included by calculating the number of bank branches within a radius of 5 km of a resource enterprise (*bank_5*). To ensure robustness, we also consider radiuses of 10 km and 20 km (*bank_10*, *bank_20*). The geographic coordinates of resource enterprises and bank branches are obtained using Baidu Maps, one of the most widely used digital maps in China. The obtained geographical coordinates from Baidu Maps are augmented with Python-collected information from the CSMAR Database and the financial license information database of the China Banking Regulatory Commission (https://xkz.cbirc.gov.cn/jr/, accessed on 10 March 2022).

Inspired by Fabrizi et al. (2018) [75] and Jia et al. (2019) [76], we use the number of green patents as a measure of resource enterprises’ green innovation level. Enterprises’ patent data come from and China National Intellectual Property Administration, (https://pss-system.cponline.cnipa.gov.cn/conventionalSearch, accessed on 10 March 2022) respectively. Specifically, we identify green patents as those that fall within the scope of the International Patent Classification (IPC) Green Inventory (https://www.wipo.int/classifications/ipc/en/green_inventory, accessed on 28 March 2022).

As for control variables, this study considers the following ones which may influence financial deepening and/or green innovation at the enterprise level: enterprise profitability (*roa*; measured by the ratio of net profit to total assets), financial leverage (*lev*; measured by the ratio of total liabilities to total assets), cash flow (*cash*; measured by the ratio of net cash flow from operating activities to total liabilities), leadership structure (*dual*; a dummy assigned a value of 1 if the enterprise’s chairman and general manager are the same person, otherwise 0), size of the board of directors (*bsize*; measured by the number of board members plus 1, logarithmic value), growth rate (*grow*; growth rate of fixed assets), proportion of independent directors (*indep*; measured by the ratio of independent directors to the total number of board members), and market value (*tq*; measured by Tobin Q) [60,77]. The data used for measuring those controls are from the CSMAR Database and the Wind Economic Database. Monetary items are measured in 2007 constant prices.

#### 3.3.3. Descriptive Statistics

The details measurements and descriptive statistics of variables are shown in Table 1. The maximum and minimum logarithmic values of city-level carbon emissions are 14.65 and 9.95, respectively. This indicates that there are significant differences among Chinese cities in terms of carbon emissions. On average, cities in our sample generate less than 0.06 green patent per 100 employees. The average financial deepening level is about 0.06 branch per km^2^. Other control variables indicate considerably regional heterogeneity.

In terms of enterprise-level data, the maximum, minimum and mean values of green innovation level are 6.0162, 0 and 0.4945, respectively. This indicates the large gap in terms of green innovation level among the resource enterprises. The mean values of enterprise financial development variables (*bank_5*, *bank_10*, *bank_20*) are 3.4507, 4.3147 and 5.0108, respectively. This means that the number of bank branches around the enterprise increases gradually with the expansion of the investigation radius.

## 4. Results and Discussion

### 4.1. Effect of Financial Deepening on Carbon Emissions

We first examine the impact of financial deepening on carbon emissions using the complied city-level data. The estimates on the effects corresponding to Equation (1) are shown in column (1) in Table 2. Given that there are considerable heterogeneities among China’s cities in terms of institution and socioeconomic contexts, we also control city- and year-fixed effects and city-level characteristics to capture the regional factors at the city level.

The results indicate that the estimate of *FD* is significantly negative at the 1% level, implying that financial deepening contributes to city-level carbon reductions, as shown by the considerably decreased CO_2_ emissions. Hypothesis 1b is thus verified. This result is consistent with Acheampong (2019) [33] and Dong (2022) [45], who argued that financial development has a dampening effect on carbon emissions. It suggests that, as financial development continues deepening, financing constraints on microeconomic entities are easing, contributing to increased green research and development (R&D) investments. Through improving green technology, both energy efficiency and renewable energy consumption can be promoted, ultimately achieving the goal of improving resource utilization and reducing carbon emissions. As for controls, the estimated coefficients of *pgdp*, *urb* and *es* are significantly positive at the 1% level, arguing that the level of economic development, the level of urbanization and energy structure positively affect carbon emissions, which are consistent with existing studies focusing on China [6,69].

### 4.2. Checks of the Green Innovation Channel

We further identify the channels for the identified impact of financial deepening on carbon emissions. As discussed above, we focus on the role of green innovation in this nexus. The estimates on the role corresponding to Equation (2) are shown in column (2) in Table 2. City- and year-fixed effects and city-level characteristics are also controlled to capture the city-level factors. The results indicate that the estimate of *FD* is significantly positive at the 1% level, implying that financial deepening promotes city-level green innovation, as shown by the considerably increased number of green patents per 100 employees. Hypothesis 2a is thus verified.

As for controls, the estimated coefficients of *pgdp* and *es* are significantly negative, while the estimates of *pop*, *urb* and *citywage* are significantly positive. The results for energy structure, population size, the level of urbanization and wage level are generally in line with the literature [78,79,80]. Notably, there is a significant negative relationship between the levels of economic development and green innovation, implying that economic development hinders green innovation in China’s cities, which is different from Yu et al. (2021) [50], who argue a positive nexus. One possible explanation is the threshold effect of economic development [81]; before the economy reaches a certain point, the increase in the per capita GDP is likely to discourage environmental improvements activities such as green innovation. However, after the economy develops to a certain level, the increase in the per capita GDP would stimulate environmental improvements activities. The level of economic development in most Chinese cities is still much lower than the level in developed economies, and even lower than the global average (i.e., in 2020, the per capita GDP in most Chinese cities was still less than 10,000 U.S. dollars, while the average values for the world and OECD economies were 10,919 and 38,116 U.S. dollars, respectively. (https://www.statista.com/statistics/268751/global-gross-domestic-product-gdp-per-capita/; https://data.worldbank.org/indicator/NY.GDP.PCAP.CD?locations=OE, accessed on 9 July 2022), which may inhibit China’s progress in green innovation [82].

### 4.3. Addressing Endogeneity

In literature, there are potential reverse causality or endogeneity concerns in terms of the environmental quality and financial deepening relationship [34,83,84]. Besides, many factors (i.e., technological level, FDI) may affect carbon emissions [63,85,86], which means that excluding these factors might result in omitted variable bias. Given that, the simultaneous endogenous problem between financial deepening and carbon emissions may exist. To address the endogeneity concerns, the 2SLS regression method is employed [69,87].

Specifically, we use the number of gates of city walls in the Qing Dynasty (1644–1911) as an IV to instrument for financial deepening. For thousands of years, walls were built around cities mainly for defensive purposes in China. A city’s boundaries had been clearly defined by the city walls, and the number of gates of city walls was positively correlated with city population, size and economic level [88,89], which makes the number of gates of city walls relevant for contemporary financial deepening level. On the other hand, city walls in the Qing Dynasty were built several hundreds of years ago, which implies that the number of gates of city walls is highly likely to satisfy the exclusionary restriction for a valid IV. The city wall gates data are from the G.W. Skinner Data Archive, Harvard University (https://dataverse.harvard.edu/dataverse/hrs, accessed on 1 May 2022). The results are shown in Table 3.

Considering the identified IV, the 2SLS method is used to re-estimate the baseline model. According to the results in column (1), the coefficient of the IV is significantly positive at the 1% level. The results of the Underidentification LM statistic and the C-D Wald test indicate that the IV satisfies the correlation characteristics and there is no weak instrumental variable issue. The estimates in column (2) indicate that financial deepening imposed significant and negative impacts on carbon emissions.

### 4.4. Robustness Tests

To further verify the reliability of the above estimates, this study conducts a series of robustness tests.

Replacing the key variables. One might be concerned about the precision of the ways in which we measure key variables. Inspired by Baele et al. (2014) [90], Eskander and Fankhauser (2020) [91] and Lee and Luca (2019) [92], we use CO_2_ emissions per unit of GDP as a proxy for carbon emissions (*rco2*) and the ratio of total loans of financial institutions to the size of the city to measure financial deepening (*fin*). The results are presented in columns (1) and (2) in Table 4, which remain robust.

Considering provincial differences. In China, cities in provinces with better environmental regulations and favorable ecological conditions are more likely to stimulate the development of the services sectors, including the financial sector (i.e., some southern coastal provinces including Hainan and Guangdong). Hence, we also control province-fixed effects to capture the regional factors at the provincial level. The results presented in column (3) in Table 4 further prove the robustness of our estimates.

Considering the impact of other policies. The impact on carbon emissions may be due to the effects of other policies. In this case, the benchmark estimates would be biased. In China, municipality, provincial capitals and cities specifically designated in the state plan were selected as cities listed as having key environmental protection, which have been specifically regulated for preventing and controlling atmospheric pollution (http://www.npc.gov.cn/zgrdw/npc/flsyywd/xingzheng/2002-07/11/content_297385.htm, accessed on 15 June 2022). This environmental policy may have a negative impact on city-level carbon emissions. To exclude the influence of this policy, we exclude municipality, provincial capitals and cities specifically designated in the state plan from the estimated sample. The results are shown in column (4) of Table 4 and support the robustness of the benchmark estimates.

Considering the lagged effect. The impact of financial deepening on carbon emissions may be lagged. Inspired by Moody (2001) [93], we consider both carbon emissions lagged one period (*Lco2*) and those lagged two periods (*L2co2*) and report the results in columns (5) and (6) in Table 4. The results also support that the previous results are robust.

### 4.5. Further Analysis Using Enterprise-Level Data

The previous estimates are conducted at the city level. Since resource enterprises have been identified as a major carbon emitter in China’s cities [57,58,59,60], it is useful to explore the above-identified nexus using resource enterprises’ data. The enterprise-level empirical strategy used is similar to the above city-level analysis. Considering that the carbon emissions data of resource enterprises are unavailable, the enterprise-level analysis mainly empirically explores the role of green innovation in the identified nexus.

The empirical estimates are presented in Table 5. The results in columns (1)–(4) indicate that that the estimate of *bank_5* is significantly positive at the 1% level. The results in columns (5) and (6) show that the estimates of *bank_10* and *bank_20* are both significantly positive at the 1% level. These consistently imply that financial deepening promotes resource enterprises’ green innovation. Hence, Hypothesis 2a is further verified.

For China’s resource enterprises, the long investment return period and high uncertainty of green innovation mean that those enterprises need continuous and considerable amounts of investment [53,94]. Therefore, it is difficult to rely on the resource enterprise’s own funds for green innovation, implying that external resources are in demand for alleviating the financing constraints of green innovation activities. Financial deepening provides opportunities to meet the continuous and considerable funds needed with less costs, which can therefore alleviate the financing constraints and stimulate resource enterprises’ green innovation.

## 5. Conclusions

Using newly compiled city-level (287 prefecture-level and above cities) and enterprise-level (resource enterprises listed on the Chinese A-shares) datasets, this study addresses the effect of financial deepening on carbon emissions in China, with a particular consideration of green innovation.

The empirical analysis shows that financial deepening contributes to carbon reductions, as shown by the considerably decreased CO_2_ emissions. Both the city-level and enterprise-level estimates support that financial deepening promotes green innovation. Promoting green innovation is identified as an important mechanism through which financial deepening can contribute to carbon reductions. The 2SLS method is adopted to effectively solve the endogenous problem. These results are robust across a serious of robustness checks.

Policy implications are proposed based on our empirical estimates. First, cities’ policymakers should strengthen the guidance of policies related to financial deepening to promote carbon reductions. For example, cities’ policymakers are suggested to strengthen the cooperation between the authorities and financial institutions. The authorities could play a guiding role in developing financial deepening, considering both the applicability of local conditions and the development of resource enterprises. Besides, financial institutions should actively ease the credit constraints of resource enterprises and promote the optimization of financial resource allocation. Second, green innovation plays an important role in the development of financial institutions and carbon reductions. Policymakers are encouraged to continue optimizing the marketization, legalization and international business environment and improving the allocation efficiency of factor markets to better empower green innovation. Policymakers are also encouraged to promote resource enterprises’ green innovation through effective subsidies and tax breaks, further alleviating the financing constraints of green innovation activities.

Our study has some limitations. The first is that the carbon emissions data of China’s resource enterprises are unavailable, which limits the direct enterprise-level estimation about the nexus between financial deepening and carbon emissions. Future research could pay attention to using available enterprise-level emissions data to further explore the identified nexus. The second is that our conclusions might be impacted by China’s unique sociocultural and sociopolitical contexts. We may pay attention in future research to cross-national contexts to contribute more generalized knowledge.

## Figures and Tables

**Table 1:** Descriptive statistics.

Variables	Definitions	Mean	Std. Dev
City carbon emissions (*co*2)	CO_2_ emissions from cities (tons, logarithm)	12.2658	0.7679
City green innovation level (*green*)	Number of green patents per 100 employees	0.0564	0.0560
City-level financial deepening (*FD*)	Number of bank branches to the size of the city	0.0595	0.0428
Level of economic development (*pgdp*)	GDP per capita (yuan/person, logarithm)	10.4767	0.6882
Population size (*pop*)	Population (person, logarithm)	5.8744	0.6948
Level of industrialization (*ind*)	The ratio of the secondary industry output value to GDP (%)	48.3813	12.1920
Level of urbanization (*urban*)	The ratio of urban area population to the city’s total population (%)	52.9379	10.0714
Energy structure (*es*)	The coal share of total primary energy supply (%)	44.54	13.17
Wage level (*citywage*)	The average salary of the city’s employees (yuan, logarithm)	4.6142	2.0202
Consumption level (*rsc*)	The total retail sales of social consumer goods (yuan, logarithm)	15.2952	1.1250
Enterprise-level financial deepening (*bank_5*)	The number of bank branches within a radius of 5 km of a resource enterprise	3.4507	1.9671
Enterprise-level financial deepening (*bank_10*)	The number of bank branches within a radius of 10 km of a resource enterprise	4.3147	2.1879
Enterprise-level financial deepening (*bank_20*)	The number of bank branches within a radius of 20 km of a resource enterprise	5.0108	2.3677
Enterprise green innovation level (*patent*)	The number of green patents (logarithm)	0.4945	0.8331
Enterprise profitability (*roa*)	The ratio of net profit to total assets	0.0410	0.0658
Financial leverage (*lev*)	The ratio of total liabilities to total assets	0.4341	0.2096
Cash flow (*cash*)	The ratio of net cash flow from operating activities to total liabilities	0.2284	0.3381
Leadership structure (*dual*)	A dummy assigned a value of 1 if the enterprise’s chairman and general manager are the same person, otherwise 0	0.2220	0.4156
Size of the board of directors (*bsize*)	The number of board members plus 1 (logarithm)	2.2779	0.1807
Growth rate (grow)	Growth rate of fixed assets	0.1708	0.5198
Proportion of independent directors (*indep*)	The ratio of independent directors to the total number of board members	0.3695	0.0508
Market value (*tq*)	Tobin Q value	2.0602	1.3542

**Table 2:** Results on carbon emissions and green innovation at the city level.

	(1)	(2)
Variables	*co2*	*Green*
*FD*	−0.8114 ***	0.6491 ***
	(0.2382)	(0.2220)
*Pgdp*	0.0710 ***	−0.0227 **
	(0.0229)	(0.0099)
*Pop*	0.0764	0.1721 ***
	(0.0881)	(0.0539)
*Ind*	0.0009	0.0004
	(0.0006)	(0.0003)
*Urb*	0.0196 ***	0.0031 **
	(0.0031)	(0.0014)
*Es*	0.5017 ***	−0.0593 *
	(0.1391)	(0.0341)
*Citywage*	0.0109	0.0398 ***
	(0.0116)	(0.0062)
*Rsc*	0.0113	0.0030
	(0.0110)	(0.0038)
Constant	9.6059 ***	−1.1362 ***
	(0.6261)	(0.3443)
Adjusted R^2^	0.9864	0.7757
Year-fixed effects	YES	YES
City-fixed effects	YES	YES

Notes: (1) Standard errors clustered at the city level are in parentheses; (2) *** *p* < 0.01, ** *p* < 0.05, * *p* < 0.1.

**Table 3:** IV estimation results.

	(1)	(2)
Variables	*FD*	*co2*
*FD*		−1.8306 ***
		(0.3703)
*IV*	0.2465 ***	
	(0.0227)	
*pgdp*	0.0021	0.0678 ***
	(0.0028)	(0.0257)
*pop*	0.0228	0.0774
	(0.0667)	(0.1442)
*ind*	0.0001	0.0004
	(0.0001)	(0.0007)
*urb*	−0.0011 *	0.0179 ***
	(0.0007)	(0.0036)
*es*	0.0259 *	0.6108 ***
	(0.0133)	(0.1940)
*citywage*	0.0024	0.0088 **
	(0.0022)	(0.0137)
*rsc*	−0.0040	0.0329
	(0.0033)	(0.0319)
Underidentification LM statistic (*p* value)	0.000 ***	0.000 ***
C-D Wald F statistic	1611.14 ***	1611.141 ***
Year-fixed effects	YES	YES
City-fixed effects	YES	YES

Notes: (1) Standard errors clustered at the city level are in parentheses; (2) *** *p* < 0.01, ** *p* < 0.05, * *p* < 0.1.

**Table 4:** Robustness checks.

	(1)	(2)	(3)	(4)	(5)	(6)
Variables	rco2	co2	co2	co2	Lco2	L2co2
*FD*	−1.3539 ***		−0.8114 ***	−0.9672 ***	−0.8474 ***	−0.8831 ***
	(0.4662)		(0.2391)	(0.2012)	(0.2183)	(0.2200)
*fin*		−0.0132 ***				
		(0.0046)				
*pgdp*	−0.2474 ***	−0.2670 ***	0.0710 ***	0.0674 ***	0.0590 ***	0.0403 ***
	(0.0861)	(0.0885)	(0.0230)	(0.0228)	(0.0187)	(0.0143)
*pop*	−0.2191	−0.0804	0.0764	0.0352	0.0153	−0.0282
	(0.1972)	(0.1911)	(0.0884)	(0.0877)	(0.0723)	(0.0639)
*ind*	−0.0003	−0.0004	0.0009	0.0008	0.0013 **	0.0019 ***
	(0.0036)	(0.0037)	(0.0006)	(0.0006)	(0.0005)	(0.0005)
*urb*	0.0059	0.0034	0.0196 ***	0.0180 ***	0.0163 ***	0.0153 ***
	(0.0085)	(0.0092)	(0.0031)	(0.0027)	(0.0027)	(0.0025)
*es*	0.1639	0.2180	0.5017 ***	0.3989 ***	0.4534 ***	0.4753 ***
	(0.2476)	(0.2488)	(0.1397)	(0.1343)	(0.1185)	(0.1086)
*citywage*	−0.0162	0.0083	0.0109	0.0216 **	0.0062	0.0009
	(0.0176)	(0.0193)	(0.0117)	(0.0104)	(0.0098)	(0.0081)
*rsc*	−0.0498	−0.0478	0.0113	0.0124	0.0064	0.0008
	(0.0392)	(0.0393)	(0.0110)	(0.0112)	(0.0082)	(0.0062)
Constant	4.9697 ***	4.3000 ***	9.6059 ***	9.8678 ***	10.3432 ***	10.9096 ***
	(1.5038)	(1.4813)	(0.6286)	(0.5982)	(0.5059)	(0.4409)
Adjusted R^2^	0.7005	0.7033	0.9863	0.9858	0.9894	0.9914
Year-fixed effects	YES	YES	YES	YES	YES	YES
City-fixed effects	YES	YES	YES	YES	YES	YES
Province-fixed effects	NO	NO	YES	NO	NO	NO

Notes: (1) Standard errors clustered at the city level are in parentheses; (2) *** *p* < 0.01, ** *p* < 0.05.

**Table 5:** Estimates using resource enterprises’ data.

Variables	(1)	(2)	(3)	(4)	(5)	(6)
*bank_5*	0.0291 ***	0.0217 ***	0.0284 ***	0.0217 ***		
	(0.0041)	(0.0039)	(0.0041)	(0.0040)		
*bank_10*					0.0192 ***	
					(0.0036)	
*bank_20*						0.0171 ***
						(0.0033)
*roa*	0.7600 ***	0.7775 ***	0.7343 ***	0.7530 ***	0.7545 ***	0.7560 ***
	(0.1169)	(0.1139)	(0.1171)	(0.1141)	(0.1142)	(0.1141)
*lev*	0.4459 ***	0.5906 ***	0.4332 ***	0.5847 ***	0.5897 ***	0.5934 ***
	(0.0393)	(0.0390)	(0.0407)	(0.0403)	(0.0405)	(0.0406)
*cash*	0.0238	0.0231	0.0162	0.0168	0.0186	0.0179
	(0.0203)	(0.0201)	(0.0204)	(0.0202)	(0.0202)	(0.0202)
*dual*	−0.0687 ***	−0.1020 ***	−0.0581 ***	−0.0926 ***	−0.0942 ***	−0.0962 ***
	(0.0161)	(0.0160)	(0.0161)	(0.0160)	(0.0160)	(0.0160)
*bsize*	0.2265 ***	0.3815 ***	0.1892 ***	0.3508 ***	0.3521 ***	0.3542 ***
	(0.0559)	(0.0541)	(0.0551)	(0.0532)	(0.0532)	(0.0532)
*grow*	−0.0640 ***	−0.0438 ***	−0.0661 ***	−0.0460 ***	−0.0465 ***	−0.0475 ***
	(0.0109)	(0.0109)	(0.0111)	(0.0110)	(0.0110)	(0.0110)
*indep*	0.6691 ***	0.6203 ***	0.5973 ***	0.5573 ***	0.5701 ***	0.5833 ***
	(0.1840)	(0.1737)	(0.1839)	(0.1734)	(0.1736)	(0.1736)
*tq*	−0.0811 ***	−0.0780 ***	−0.0794 ***	−0.0767 ***	−0.0769 ***	−0.0767 ***
	(0.0046)	(0.0047)	(0.0047)	(0.0048)	(0.0048)	(0.0048)
Constant	−0.4719 ***	−1.2867 ***	−0.0969	−0.9378 ***	−0.9509 ***	−0.9615 ***
	(0.1723)	(0.1678)	(0.1761)	(0.1708)	(0.1709)	(0.1711)
Observations	9938	9938	9938	9938	9938	9938
Adjusted R^2^	0.0548	0.1270	0.0615	0.1335	0.1334	0.1332
Year-fixed effects	NO	YES	NO	YES	YES	YES
Industry-fixed effects	NO	NO	YES	YES	YES	YES

Notes: (1) Standard errors clustered at the enterprise level are in parentheses; (2) *** *p* < 0.01.

## Data Availability

The data presented in this study are available on reasonable request from the corresponding author.

## References

[1] IPCC (2021). Climate Change 2021: The Physical Science Basis. Contribution of Working Group I to the Sixth Assessment Report of the Intergovernmental Panel on Climate Change.

[2] Tang K., Wang M., Zhou D. (2021). Abatement potential and cost of agricultural greenhouse gases in Australian dryland farming system. Environ. Sci. Pollut. Res..

[3] Tang K., Hailu A. (2020). Smallholder farms’ adaptation to the impacts of climate change: Evidence from China’s Loess Plateau. Land Use Policy.

[4] Zhou D., Liang X., Zhou Y., Tang K. (2020). Does emission trading boost carbon productivity? Evidence from China’s pilot emission trading scheme. Int. J. Environ. Res. Public Health.

[5] Tang K., Ma C. The cost-effectiveness of agricultural greenhouse gas reduction under diverse carbon policies in China. China Agric. Econ. Rev..

[6] Tang K., Liu Y., Zhou D., Qiu Y. (2021). Urban carbon emission intensity under emission trading system in a developing economy: Evidence from 273 Chinese cities. Environ. Sci. Pollut. Res..

[7] Wu J., Ma C., Tang K. (2019). The static and dynamic heterogeneity and determinants of marginal abatement cost of CO_2_ emissions in Chinese cities. Energy.

[8] Wu J., Feng Z., Tang K. (2021). The dynamics and drivers of environmental performance in Chinese cities: A decomposition analysis. Environ. Sci. Pollut. Res..

[9] Yuan F., Tang K., Shi Q., Qiu W., Wang M. (2022). Rural women and chemical fertiliser use in rural China. J. Clean. Prod..

[10] Lin K.C., Purra M.M. (2019). Transforming China’s electricity sector: Politics of institutional change and regulation. Energy Policy.

[11] Ahlstrom D., Arregle J.L., Hitt M.A., Qian G., Ma X., Faems D. (2020). Managing technological, sociopolitical, and institutional change in the new normal. J. Manag. Stud..

[12] Tang K., He C., Ma C., Wang D. (2019). Does carbon farming provide a cost-effective option to mitigate GHG emissions? Evidence from China. Aust. J. Agric. Resour. Econ..

[13] Tang K., Hailu A., Yang Y. (2020). Agricultural chemical oxygen demand mitigation under various policies in China: A scenario analysis. J. Clean. Prod..

[14] Campiglio E., Dafermos Y., Monnin P., Ryan-Collins J., Schotten G., Tanaka M. (2018). Climate change challenges for central banks and financial regulators. Nat. Clim. Chang..

[15] Rongwei X., Xiaoying Z. (2020). Is financial development hampering or improving the resource curse? New evidence from China. Resour. Policy.

[16] Yıldırım S., Gedikli A., Erdoğan S., Yıldırım D.Ç. (2020). Natural resources rents-financial development nexus: Evidence from sixteen developing countries. Resour. Policy.

[17] Shahbaz M., Topcu B.A., Sarıgül S.S., Vo X.V. (2021). The effect of financial development on renewable energy demand: The case of developing countries. Renew. Energy.

[18] Ho C.Y., Huang S., Shi H., Wu J. (2018). Financial deepening and innovation: The role of political institutions. World Dev..

[19] Knight M. (1998). Developing countries and the globalization of financial markets. World Dev..

[20] Slesman L., Baharumshah A.Z., Azman-Saini W.N.W. (2019). Political institutions and finance-growth nexus in emerging markets and developing countries: A tale of one threshold. Q. Rev. Econ. Financ..

[21] Chen K., Long H., Liao L., Tu S., Li T. (2020). Land use transitions and urban-rural integrated development: Theoretical framework and China’s evidence. Land Use Policy.

[22] Zhang X.Q. (2016). The trends, promises and challenges of urbanisation in the world. Habitat Int..

[23] Hamnett C. (2020). Is Chinese urbanisation unique?. Urban Stud..

[24] Chong T.T.L., Lu L., Ongena S. (2013). Does banking competition alleviate or worsen credit constraints faced by small-and medium-sized enterprises? Evidence from China. J. Bank. Financ..

[25] Hou L., Hsueh S.C., Zhang S. (2020). Does formal financial development crowd in informal financing? Evidence from Chinese private enterprises. Econ. Model..

[26] Zhang L., Hsu S., Xu Z., Cheng E. (2020). Responding to financial crisis: Bank credit expansion with Chinese characteristics. China Econ. Rev..

[27] Hasan I., Wachtel P., Zhou M. (2009). Institutional development, financial deepening and economic growth: Evidence from China. J. Bank. Financ..

[28] Sehrawat M., Giri A.K. (2018). The impact of financial development, economic growth, income inequality on poverty: Evidence from India. Empir. Econ..

[29] Nasir M.A., Huynh T.L.D., Tram H.T.X. (2019). Role of financial development, economic growth foreign direct investment in driving climate change: A case of emerging ASEAN. J. Environ. Manag..

[30] Zhang Y.J. (2011). The impact of financial development on carbon emissions: An empirical analysis in China. Energy Policy.

[31] Boutabba M.A. (2014). The impact of financial development, income, energy and trade on carbon emissions: Evidence from the Indian economy. Econ. Model..

[32] Shahzad S.J.H., Kumar R.R., Zakaria M., Hurr M. (2017). Carbon emission, energy consumption, trade openness and financial development in Pakistan: A revisit. Renew. Sustain. Energy Rev..

[33] Acheampong A.O. (2019). Modelling for insight: Does financial development improve environmental quality?. Energy Econ..

[34] Acheampong A.O., Amponsah M., Boateng E. (2020). Does financial development mitigate carbon emissions? Evidence from heterogeneous financial economies. Energy Econ..

[35] Kirikkaleli D., Güngör H., Adebayo T.S. (2022). Consumption-based carbon emissions, renewable energy consumption, financial development and economic growth in Chile. Bus. Strategy Environ..

[36] Chen Y., Cheng L., Lee C.C., Wang C.S. (2021). The impact of regional banks on environmental pollution: Evidence from China’s City commercial banks. Energy Econ..

[37] Liu Z., Deng Z., He G., Wang H., Zhang X., Lin J., Qi Y., Liang X. (2022). Challenges and opportunities for carbon neutrality in China. Nat. Rev. Earth Environ..

[38] Guan D., Meng J., Reiner D.M., Zhang N., Shan Y., Mi Z., Shao S., Liu Z., Zhang Q., Davis S.J. (2018). Structural decline in China’s CO_2_ emissions through transitions in industry and energy systems. Nat. Geosci..

[39] Long C., Zhang X. (2011). Cluster-based industrialization in China: Financing and performance. J. Int. Econ..

[40] Kahouli B. (2017). The short and long run causality relationship among economic growth, energy consumption and financial development: Evidence from South Mediterranean Countries (SMCs). Energy Econ..

[41] Gaies B., Kaabia O., Ayadi R., Guesmi K., Abid I. (2019). Financial development and energy consumption: Is the MENA region different?. Energy Policy.

[42] Qin L., Hou Y., Miao X., Zhang X., Rahim S., Kirikkaleli D. (2021). Revisiting financial development and renewable energy electricity role in attaining China’s carbon neutrality target. J. Environ. Manag..

[43] Tamazian A., Chousa J.P., Vadlamannati K.C. (2009). Does higher economic and financial development lead to environmental degradation: Evidence from BRIC countries. Energy Policy.

[44] Li P., Ouyang Y. (2020). Technical change and green productivity. Environ. Resour. Econ..

[45] Dong F., Li Y., Gao Y., Zhu J., Qin C., Zhang X. (2022). Energy transition and carbon neutrality: Exploring the non-linear impact of renewable energy development on carbon emission efficiency in developed countries. Resour. Conserv. Recycl..

[46] Du K., Li P., Yan Z. (2019). Do green technology innovations contribute to carbon dioxide emission reduction? Empirical evidence from patent data. Technol. Forecast. Soc. Chang..

[47] Razzaq A., Fatima T., Murshed M. (2021). Asymmetric effects of tourism development and green innovation on economic growth and carbon emissions in Top 10 GDP Countries. J. Environ. Plan. Manag..

[48] Yuan B., Li C., Yin H., Zeng M. (2022). Green innovation and China’s CO_2_ emissions–the moderating effect of institutional quality. J. Environ. Plan. Manag..

[49] Owen R., Brennan G., Lyon F. (2018). Enabling investment for the transition to a low carbon economy: Government policy to finance early stage green innovation. Curr. Opin. Environ. Sustain..

[50] Yu C.H., Wu X., Zhang D., Chen S., Zhao J. (2021). Demand for green finance: Resolving financing constraints on green innovation in China. Energy Policy.

[51] Wang H., Qi S., Zhou C., Zhou J., Huang X. (2022). Green credit policy, government behavior and green innovation quality of enterprises. J. Clean. Prod..

[52] Abdullah M., Zailani S., Iranmanesh M., Jayaraman K. (2016). Barriers to green innovation initiatives among manufacturers: The Malaysian case. Rev. Manag. Sci..

[53] Xiang X., Liu C., Yang M. (2022). Who is financing corporate green innovation?. Int. Rev. Econ. Financ..

[54] Khan M., Ozturk I. (2021). Examining the direct and indirect effects of financial development on CO_2_ emissions for 88 developing countries. J. Environ. Manag..

[55] Hsu P.H., Tian X., Xu Y. (2014). Financial development and innovation: Cross-country evidence. J. Financ. Econ..

[56] Lv C., Shao C., Lee C.C. (2021). Green technology innovation and financial development: Do environmental regulation and innovation output matter?. Energy Econ..

[57] Liu Z. (2016). China’s Carbon Emissions Report 2016.

[58] Wang H., Lu X., Deng Y., Sun Y., Nielsen C.P., Liu Y., Zhu G., Bu M., Bi J., McElroy M.B. (2019). China’s CO_2_ peak before 2030 implied from characteristics and growth of cities. Nat. Sustain..

[59] Shan Y., Huang Q., Guan D., Hubacek K. (2020). China CO_2_ emission accounts 2016–2017. Sci. Data.

[60] Chen X., Chen G., Lin M., Tang K., Ye B. (2022). How does anti-corruption affect enterprise green innovation in China’s energy-intensive industries?. Environ. Geochem. Health.

[61] Bollen K.A., Brand J.E. (2010). A general panel model with random and fixed effects: A structural equations approach. Soc. Forces.

[62] Bernini C., Brighi P. (2018). Bank branches expansion, efficiency and local economic growth. Reg. Stud..

[63] Tang K., Zhou Y., Liang X., Zhou D. (2021). The effectiveness and heterogeneity of carbon emissions trading scheme in China. Environ. Sci. Pollut. Res..

[64] Xiao H., Zhao W., Shan Y., Guan D. (2021). CO_2_ emission accounts of Russia’s constituent entities 2005–2019. Sci. Data.

[65] Hasan I., Jackowicz K., Kowalewski O., Kozłowski Ł. (2017). Do local banking market structures matter for SME financing and performance? New evidence from an emerging economy. J. Bank. Financ..

[66] Berrone P., Fosfuri A., Gelabert L., Gomez-Mejia L.R. (2013). Necessity as the mother of ‘green’inventions: Institutional pressures and environmental innovations. Strateg. Manag. J..

[67] Consoli D., Marin G., Marzucchi A., Vona F. (2016). Do green jobs differ from non-green jobs in terms of skills and human capital?. Res. Policy.

[68] Wu J., Xu H., Tang K. (2021). Industrial agglomeration, CO_2_ emissions and regional development programs: A decomposition analysis based on 286 Chinese cities. Energy.

[69] Xu L., Fan M., Yang L., Shao S. (2021). Heterogeneous green innovations and carbon emission performance: Evidence at China’s city level. Energy Econ..

[70] Tang K., Xiong C., Wang Y., Zhou D. (2021). Carbon emissions performance trend across Chinese cities: Evidence from efficiency and convergence evaluation. Environ. Sci. Pollut. Res..

[71] Chay J.B., Park S.H., Kim S., Suh J. (2015). Financing hierarchy: Evidence from quantile regression. J. Corp. Financ..

[72] Beck T., Ongena S., Şendeniz-Yüncü İ. (2019). Keep walking? Geographical proximity, religion, and relationship banking. J. Corp. Financ..

[73] Brown M., Guin B., Kirschenmann K. (2016). Microfinance banks and financial inclusion. Rev. Financ..

[74] Skrastins J., Vig V. (2019). How organizational hierarchy affects information production. Rev. Financ. Stud..

[75] Fabrizi A., Guarini G., Meliciani V. (2018). Green patents, regulatory policies and research network policies. Res. Policy.

[76] Jia N., Huang K.G., Man Zhang C. (2019). Public governance, corporate governance, and firm innovation: An examination of state-owned enterprises. Acad. Manag. J..

[77] Tang K., Qiu Y., Zhou D. (2020). Does command-and-control regulation promote green innovation performance? Evidence from China’s industrial enterprises. Sci. Total Environ..

[78] Du K., Cheng Y., Yao X. (2021). Environmental regulation, green technology innovation, and industrial structure upgrading: The road to the green transformation of Chinese cities. Energy Econ..

[79] Lin B., Zhu J. (2021). Impact of China’s new-type urbanization on energy intensity: A city-level analysis. Energy Econ..

[80] Zhang D., Zheng M., Feng G.F., Chang C.P. (2022). Does an environmental policy bring to green innovation in renewable energy?. Renew. Energy.

[81] Song M., Tao J., Wang S. (2015). FDI, technology spillovers and green innovation in China: Analysis based on Data Envelopment Analysis. Ann. Oper. Res..

[82] Li L., Li M., Ma S., Zheng Y., Pan C. (2022). Does the construction of innovative cities promote urban green innovation?. J. Environ. Manag..

[83] Jacobs B.W., Singhal V.R., Subramanian R. (2010). An empirical investigation of environmental performance and the market value of the firm. J. Oper. Manag..

[84] Tao R., Su C.W., Naqvi B., Rizvi S.K.A. (2022). Can Fintech development pave the way for a transition towards low-carbon economy: A global perspective. Technol. Forecast. Soc. Chang..

[85] Iqbal N., Abbasi K.R., Shinwari R., Guangcai W., Ahmad M., Tang K. (2021). Does exports diversification and environmental innovation achieve carbon neutrality target of OECD economies?. J. Environ. Manag..

[86] Yuan F., Tang K., Shi Q. (2021). Does Internet use reduce chemical fertilizer use? Evidence from rural households in China. Environ. Sci. Pollut. Res..

[87] Ozturk I., Ullah S. (2022). Does digital financial inclusion matter for economic growth and environmental sustainability in OBRI economies? An empirical analysis. Resour. Conserv. Recycl..

[88] Ioannides Y.M., Zhang J. (2017). Walled cities in late imperial China. J. Urban Econ..

[89] Xing C., Zhang J. (2017). The preference for larger cities in China: Evidence from rural-urban migrants. China Econ. Rev..

[90] Baele L., Farooq M., Ongena S. (2014). Of religion and redemption: Evidence from default on Islamic loans. J. Bank. Financ..

[91] Eskander S.M., Fankhauser S. (2020). Reduction in greenhouse gas emissions from national climate legislation. Nat. Clim. Chang..

[92] Lee N., Luca D. (2019). The big-city bias in access to finance: Evidence from firm perceptions in almost 100 countries. J. Econ. Geogr..

[93] Moody C.E. (2001). Testing for the effects of concealed weapons laws: Specification errors and robustness. J. Law Econ..

[94] Cailou J., DeHai L. (2022). Does venture capital stimulate the innovation of China’s new energy enterprises?. Energy.

